# Bone Marrow Mesenchymal Stem Cells for Improving Hematopoietic Function: An In Vitro and In Vivo Model. Part 2: Effect on Bone Marrow Microenvironment

**DOI:** 10.1371/journal.pone.0026241

**Published:** 2011-10-20

**Authors:** Soraya Carrancio, Belen Blanco, Carlos Romo, Sandra Muntion, Natalia Lopez-Holgado, Juan F. Blanco, Jesus G. Briñon, Jesus F. San Miguel, Fermin M. Sanchez-Guijo, M. Consuelo del Cañizo

**Affiliations:** 1 Servicio de Hematología, Hospital Universitario de Salamanca, Salamanca, Spain; 2 Centro en Red de Medicina Regenerativa y Terapia Celular de Castilla y León and Red Nacional de Terapia Celular (Tercel, ISCIII), Castilla y León, Spain; 3 Centro de Investigación del Cáncer-IBMCC (Universidad de Salamanca-CSIC), Salamanca, Spain; 4 Servicio de Traumatología, Hospital Universitario de Salamanca, Salamanca, Spain; 5 Departamento de Biologia Celular y Patologia, Universidad de Salamanca, Spain; University of Medicine and Dentistry of New Jersey, United States of America

## Abstract

The aim of the present study was to determine how mesenchymal stem cells (MSC) could improve bone marrow (BM) stroma function after damage, both *in vitro* and *in vivo*. Human MSC from 20 healthy donors were isolated and expanded. Mobilized selected CD34^+^ progenitor cells were obtained from 20 HSCT donors. For *in vitro* study, long-term bone marrow cultures (LTBMC) were performed using a etoposide damaged stromal model to test MSC effect in stromal confluence, capability of MSC to lodge in stromal layer as well as some molecules (SDF1, osteopontin,) involved in hematopoietic niche maintenance were analyzed. For the *in vivo* model, 64 NOD/SCID recipients were transplanted with CD34+ cells administered either by intravenous (IV) or intrabone (IB) route, with or without BM derived MSC. MSC lodgement within the BM niche was assessed by FISH analysis and the expression of SDF1 and osteopontin by immunohistochemistry. *In vivo* study showed that when the stromal damage was severe, TP-MSC could lodge in the etoposide-treated BM stroma, as shown by FISH analysis. Osteopontin and SDF1 were differently expressed in damaged stroma and their expression restored after TP-MSC addition. Human *in vivo* MSC lodgement was observed within BM niche by FISH, but MSC only were detected and not in the contralateral femurs. Human MSC were located around blood vessels in the subendoestal region of femurs and expressed SDF1 and osteopontin. In summary, our data show that MSC can restore BM stromal function and also engraft when a higher stromal damage was done. Interestingly, MSC were detected locally where they were administered but not in the contralateral femur.

## Introduction

Hematopoietic stem cell transplantation (HSCT) is used to treat several disorders of the immunohematopoietic system [1]–[4]. The conditioning regimen is toxic for both hematopoietic stem cells (HSC) and BM microenvironment [5], [6]. The latter is crucial to ensure normal hematopoietic engraftment after transplantation [7]–[9]. Hematopoiesis requires the cooperation between progenitors and a variety of functionally and phenotypically different cell types that form the bone marrow (BM) stroma. Since BM stroma plays an important role in homing, engraftment, self-renewal, and differentiation of HSC, it has been postulated that stromal damage caused by conditioning regimens may have a profound influence on engraftment kinetics [10]. MSC are the stromal cell progenitors, so the addition of third party-MSC (TP-MSC) may result in better engraftment and hematopoiesis after HSCT. On the basis of this hypothesis, several clinical trials have been performed to study whether the co-infusion of MSC along with HSC could improve hematopoietic engraftment [11], [12]. The mechanisms by which the MSC exert their beneficial effect are not well understood and the capacity of MSC to engraft in recipient BM is a matter of controversy. Most studies report a lack of engraftment of donor MSC suggesting a paracrine effect [13], but they can eventually be detected [14], especially in cases where there is considerable stromal damage [15]. From a molecular point of view, SDF1 and osteopontin have been proposed as main tools of MSC to regulate hematopoietic engraftment kinetics.

In the first part of this work, we studied the effect of TP-MSC on the hematopoietic population but our purpose was also to know the mechanism involved at stromal level. The aim of the present study was to analyze, both *in vitro* and *in vivo*, how MSC can enhance hematopoiesis acting on the BM microenvironment.

## Results

**Figure 1:**
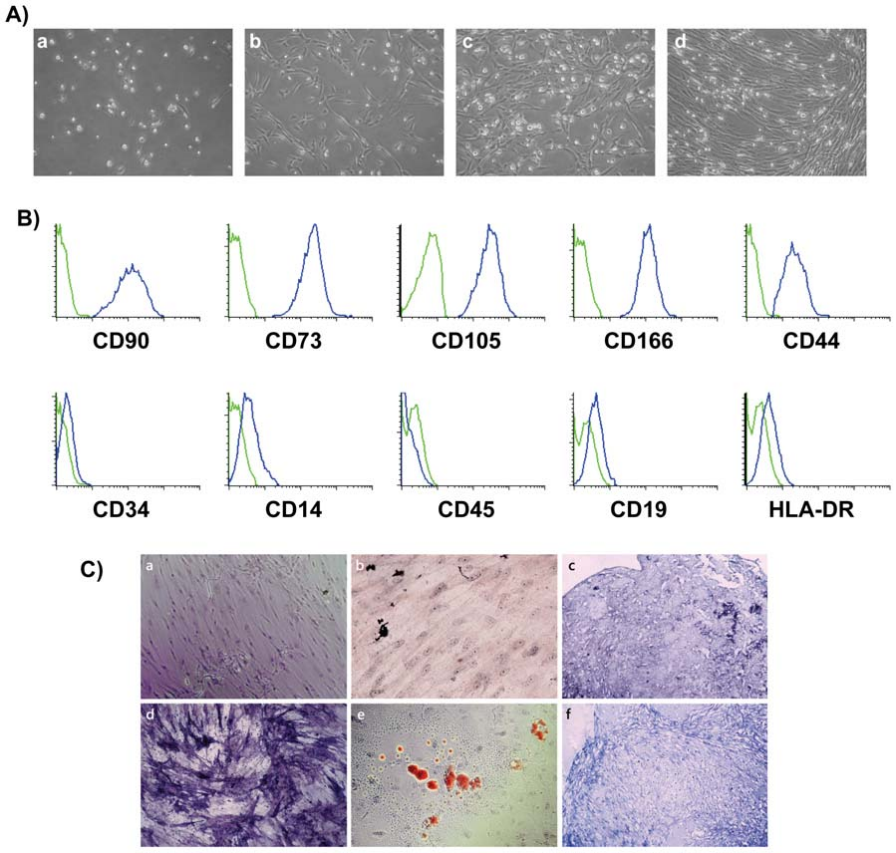
Characterization of human MSC. **A**) Images from MSC expansion. **B**) Mean fluorescence intensity of stained MSC (blue) and control non-stained MSC (green). **E**) *In vitro* differentiation of MSC to osteoblasts (a, d), adipocytes (b, e) and chondrocytes (c, f) under control conditions (a, b, c) or with differentiation medium (d, e, f).

MSC from BM samples could be expanded in all cases. All of them adhered to plastic surfaces, were capable of differentiating into adipocytes, osteoblasts and chondrocytes, expressed the antigens CD44, CD73, CD90, CD105, and CD166 and were negative for hematopoietic antigens thus fulfilling the criteria proposed for MSC definition by the International Society for Cellular Therapy (ISCT) (Figure 1) [16]. MSC expansion capacity was very variable but this variability was not related to age or gender.

### *In vitro* MSC lodging assay

To evaluate the capacity of TP-MSC to improve stromas after etoposide treatment, we assessed the percentage of confluence of the stromal layer. It was always 100% in untreated cultures, but it decreased in high-dose etoposide-treated stromas to 37% and 19% after 5 and 15 days of treatment, respectively. When treated stromas were cultured with TP-MSC supernatants, the confluence increased (40% and 46% after 5 and 15 days, respectively) and, when TP-MSC were added, the confluence improved to 100%. In the latter group, the capacity of these cells to lodge within the stromal layer was evaluated by FISH for X and Y chromosomes because TP-MSC from a mismatched-sex donor were added. Results are shown in Table 1. TP-MSC were able to lodge in etoposide-treated stromas and their percentage increased in a dose-dependent manner.

**Table 1:** *In vitro* TP-MSC-lodging capacity.

Etoposide concentration(µM)	% Donor cells	
	Day 5	Day 15
**0**	0.1	0.0
**50**	69.0	67.0
**100**	97.0	99.0

Results expressed as percentages of TP-MSC counted after FISH staining of sex chromosomes (n = 10).

### *In vitro* osteopontin and SDF-1α expression in MSC

In order to establish whether TP-MSC supernatant could improve chemotherapy-induced stromal damage, it was added to MSC that had previously been exposed to etoposide assessing SDF-1 and OPN expression in MSC. Supernatants were added to culture plates over 2 days (short-term) or 21 days (long-term).

**Figure 2:**
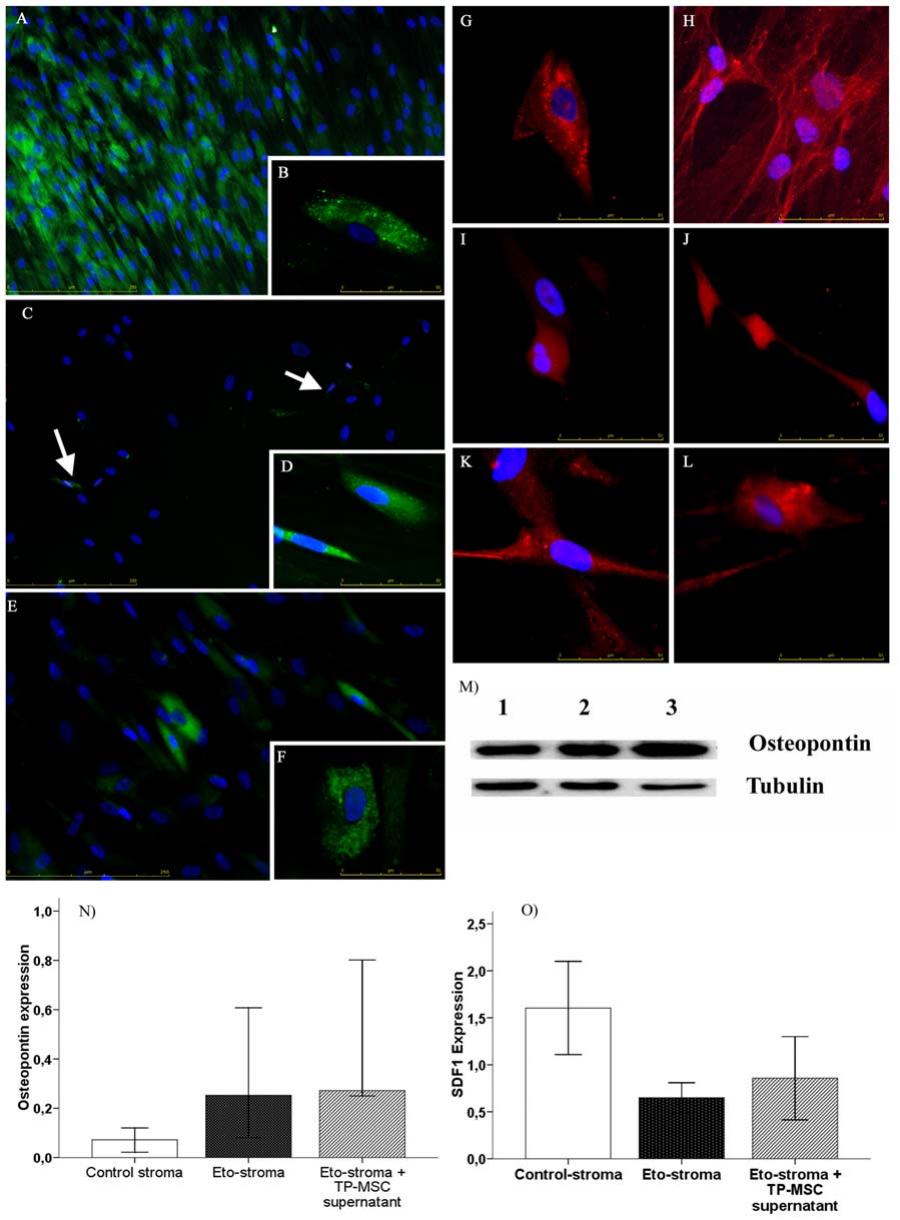
SDF-1α and osteopontin *in vitro* expression by MSC. **A**) SDF-1 expression in control stromas: positive cluster (on the left) surrounded by negative cells (right) showing a polygonal shape (**B**). **C**) In etoposide-treated stromas SDF-1 expression was weaker and cells had different shapes (**D**). **E**) In treated stromas cultured with TP-MSC supernatant, there was an increase in the number of positive cells, which were polygon-shaped (**F**). Regarding osteopontin, in control stromas it was expressed in polygonal cells (**G, H**). In etoposide-treated stromas, osteopontin expression was stronger than in controls and was located in unusually shaped cells in standard culture medium (**I, J**) and it was also high expressed in treated-stromas cultured with TP-MSC supernatant (**K, L**). **M**) As confirmed by western blot, osteopontin expression was lower in untreated stromas than in etoposide-treated stromas (1 = Control; 2 = Etoposide-treated; 3 = Etoposide-treated+TP-MSC supernatant). Finally, the expression of both molecules osteopontin (**N**) and SDF-1 (**O**) was confirmed by PCR.

Regarding SDF1 long-term exposure, control cultures showed polygonal strongly positive cells that always formed well-defined clusters surrounded by negative cells (Figure 2A, B). By contrast, very few cells with either polygonal or fibroblastic shapes were seen in treated cultures (Figure 2C, D). When TP-MSC supernatant was added to treated cultures, some small polygonal positive clusters were once again observed (Figure 2E, F).

In control assays very few cells showed osteopontin when analyzed by immunofluorescence. However, etoposide-treated stromas showed higher proportion of positive cells that showed unusual shapes with long prolongations. After three weeks, control stromas slight more polygonal cells expressing osteopontin (Figure 2G, H). By contrast, in treated cultures the number of cells expressing osteopontin was increased in both round cells as well as those showing prolongations (Figure 2I, J). This feature was more evident in treated stromas that also had received TP-MSC supernatant (Figure 2K, L).

To confirm these findings, western blot for osteopontin was performed. We found that osteopontin expression was stronger in treated cultures three weeks after etoposide-treatment, the effect being greater when cultures also received TP-MSC supernatant (Figure 2M).

RT-PCR was used to quantify the levels of osteopontin and SFD1 expression. This data confirmed the previous immunohistochemical findings. Osteopontin expression was stronger in stromal cells following etoposide treatment (after adding both medium or TP-MSC supernatant) (Figure 2N). By contrast, SDF-1 expression was decreased after etoposide stromal damage and its expression increased after adding TP-MSC supernatant (Figure 2O).

### Human MSC detection in murine BM

**Figure 3:**
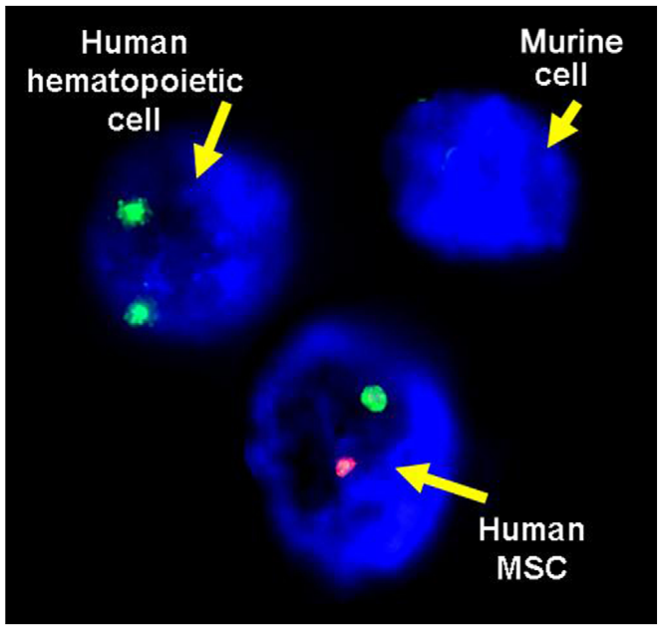
Example of human MSC detection by FISH. In this case cells were expanded from murine BM 6 weeks after transplantation human cells from sex-mismatched donors, female HSC and male MSC (green for X chromosome and red for Y chromosome).

With the strategy employed for *in vitro* lodgment analyses by FISH (using human sexual chromosomes probes since human HSC and MSC were sex-mismatched), we were able to identify three cell types within the recipient BM: murine BM cells (no signal), human HSC-derived cells and human MSC-derived cells (Figure 3). In 19 out of 32 samples of murine BM a sufficient number of MSC for FISH analysis were obtained. In all cases murine BM cells and human HSC-derived cells could be found (see Part 1). Nevertheless, we consistently found that human MSC were exclusively detected by FISH in femurs where they were previously injected, with no signal in contralateral ones (Table 2).

**Table 2:** Human *in vivo* MSC engraftment detected by FISH.

	Right femur	Left femur
**IV CD34^+^ cells + IB MSC**	22 (0–38)	-
**IB CD34^+^ cells + IB MSC**	28 (0–43)	-

Results expressed as median number of human MSC detected by slide (range).

MSC: Mesenchymal stem cells; IV: intravenous injection; IB: intrabone injection.

### Osteopontin and SDF-1α expression in murine MSC

In order to study the expression of osteopontin and SDF1α as relevant molecules involved in the *in vivo* effect of TP-MSC in hematopoietic engraftment, we analyzed their expression in femurs from previously injected mice.

**Figure 4:**
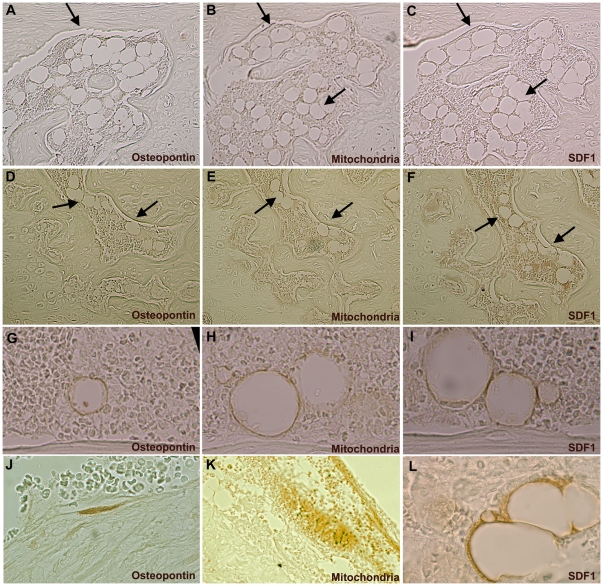
SDF-1α and osteopontin *in vivo* expression by human MSC. Human MSC expressing osteopontin and SDF1 could be found close to femur endostium or around blood vessels when they were analyzed in sequential sections (10×) (**A–F**). In most cases these human cells were positive for both osteopontin and SDF1 (**G–I**) (100×), but in some cases they were positive only for osteopontin in endostium (**J**) (100×) or SDF1 around blood vessels (**L**) (100×). Human hematopoietic cells could be detected forming colonies or groups of human mitochondria positive cells negative for osteopontin and SDF1 (**K**) (20×).

When human mitochondria were stained, we could observe that human cells were located into the BM cavity of murine femurs, mainly distributed in the epiphysis and showing a higher number close to the injected site (inthe epiphysis located close to the knee). Most human cells showed a round shape, used to locate together and were negative for osteopontin and SDF1 showing that they were human hematopoietic cells (Figure 4K). In injected femurs some cells positive for human mitochondria and for osteopontin and/or SDF1 could be found (Figure 4A–J).

Only few positive cells for osteopontin were observed and all of them were from human origin and only were present in the previously injected femurs. Osteopontin expressing cells showed fibroblastic shape when they were located in the endosteal surface of bones (Figure 4J) or a pericyte-like shape if they were around blood vessels (Figure 4A,G).

Regarding SDF1 expression by human MSC, we could confirm that in all cases, SDF1 expressing cells were from human origin as they were also positive for human mitochondria staining. Human SDF1 positive cells showed fibroblastic or polygonal shape and could be detected at two levels: some of them were distributed between hematopoietic cells or close to the endostium (Figure 4C,F), but other had a clear perivascular distribution (Figure 4G).

In most cases, human MSC located in the femur endostium, were positive for both osteopontin and SDF1, but those could show only SDF1 or both molecules SDF1 and osteopontin. These cells used to be located near blood vessels in the subendosteal region.

## Discussion

After transplantation, HSC home to the BM microenvironment where they proliferate and replace the original hematopoietic system [17]. HSC can self-renew and also give rise to differentiated cells of various lineages within the BM. Both, self-renewal and differentiation potential are crucial and tightly regulated. However, the mechanisms controlling fate decisions and *in vivo* behavior of HSC are only partially understood [18]. The microenvironment of HSC in the BM supports both the maintenance of the stem cell pool and the differentiation of HSC [7]. A balance between these processes is mediated by soluble and membrane-bound cytokines, extracellular matrix components, and direct cell-to-cell contact.

Conditioning regimens are employed pretransplant to eliminate malignant or abnormal cells and/or to immunosuppress the recipient to facilitate hematopoietic stem cell (HSC) engraftment. The conditioning regimen is known to be able to damage not only the HSC but also the bone marrow (BM) microenvironment [5], [6]. It has been suggested that stromal damage caused by conditioning regimens or previous chemo-therapy treatments may have a profound influence on engraftment kinetics [10]. BM MSC contribute to the regeneration of mesenchymal tissues such as bone, cartilage, muscle, adipose, and marrow stroma and are essential in providing support for the growth and differentiation of primitive hematopoietic cells within the BM niche [19]. They participate in the marrow stroma function producing a vast array of matrix molecules, cytokines and growth factors [7], [20]. In this line, it has been hypothesized that the addition of TP-MSC may result in better engraftment after HSCT, and several trials have examined whether the co-infusion of MSC and HSC improve this outcome [11], [21]. In a previous part of this work we have demonstrated that the addition of TP-MSC could increase hematopoiesis [22]. We have used an *in vitro* model of stromal damage based on etoposide treatment and an *in vivo* model based on the SCID-repopulating assays to test the effect of TP-MSC in hematopoietic engraftment. In the current work we also wanted to know the mechanism involved in the MSC effect at the stromal level.

The mechanisms by which the MSC exert their beneficial effect are not well understood and the capacity of MSC to engraft in recipient BM is a matter of controversy. Most studies report a lack of engraftment of donor MSC [13], but they can eventually be detected [14], especially in cases where there is considerable stromal damage [15]. The first aim of this study was to test *in vitro* whether stromal damage could favor the lodging of healthy TP-MSC in the culture by inducing different degrees of damage (modifying etoposide concentration) and we observed that TP-MSC lodging was dose-dependent (Table 1). In order to find out whether TP-MSC effect requires direct cell-to-cell contact or is performed by soluble factors through a paracrine effect, TP-MSC supernatant was added to improve the damaged stroma. This approach only produced a slight improvement, suggesting that TP-MSC contact is needed when profound stromal damage has occurred, although when the damage is moderate, the paracrine effect of TP-MSC may be enough to produce an improvement.

In order to study the mechanism involved in MSC beneficial effect, two of the most important molecules involved in the engraftment and maintenance of HPC as osteopontin (an osteoblastic molecule involved in the stem cell niche) [23] and SDF-1α (a chemokine involved in HPC trafficking and engraftment by its receptor CXCR4) [24] were analyzed. In the previous part of this work, when hematopoietic function was studied, we could observe that BM stromal damage impairs hematopoiesis at two levels: on the most immature cells (fewer cobblestone areas) and by decreasing committed progenitors (decreased production of CFU-GM) [22]. In the present work, we aimed to establish whether SDF-1 and osteopontin were involved. Some studies have shown a lack of osteopontin in MSC [25], whereas recent publications have demonstrated the relevance of this molecule as a hematopoietic support [26]. In this study a stronger activity was observed in etoposide-treated BM stroma. This feature could be interpreted as evidence of a mechanism by which the BM-damaged stroma preserve the most immature progenitors by increasing molecules involved in their maintenance. By contrast, SDF-1α expression was highly disrupted in treated stromas, suggesting that the SDF-1/CXCR4 axis is affected in damaged hematopoiesis [27], [28]. It has been previously shown that SDF-1 favors hematopoietic engraftment [29]. In our model SDF-1α expression was not homogeneous, there were some cell clusters expressing strong activity which could represent hematopoietic niches([27], [30]). The paracrine effect of TP-MSC supernatant on damaged stromas allowed a slight increase in SDF1 expression, suggesting a restoration of their hematopoietic support capability. The strongest expression of SDF-1α, which can act as a chemotactic factor, was observed in normal cultures in accordance with the stronger expression of CXCR4 in HPC described in the previous part of this work. Moreover, the addition of TP- MSC to damaged stromas could improve the secretion of SDF1α, resulting in a higher level of CXCR4 expression, thereby favoring engraftment.

In order to confirm previous *in vitro* data a xenotransplantation model was carried out. Some groups have proposed the use of MSC, in order to improve engraftment, especially in cases of high risk of graft failure such as haploidentical transplantation, allo-immunized patients or cord blood transplantation [11], [21], [31], [32]. When MSC are injected IV, most of them are retained within the lungs microvasculature. To improve MSC local effect new trials searching for new routes of administration are being explored [33], [34]. In the HSCT setting, IB injection of hematopoietic progenitors has been shown to enhance engraftment [35], [36]. We have therefore considered that this route of delivery could be optimal for MSC administration. Previous data showed that TP-MSC could increase hematopoietic engraftment but we also wanted to know the mechanism involved in MSC effect. In fact there is considerable debate about the capability of MSC to engraft in recipient BM. In a previous study performed by our own group MSC engraftment was observed after HSCT, especially in cases in which considerable stromal damage was suspected [15]. The results of MSC chimerism studies deserve an additional comment, since due to their IB injection we have detected the human MSC only in the injected femurs and in a very low proportion showing that these cells can engraft and remain for long-time when locally injected. However their low number seems not to be enough to justify benefit on engraftment based on cell to cell contact suggesting that MSC may secrete several factors that enhance HSC lodgement [37].

Also we wanted to know their spatial distribution within the BM as well as their expression of osteopontin and SDF1. Within the BM two different niches have been described: the osteoblastic and the endothelial niche. In our model, human MSC were located in both, in the femur endostium as a part of the osteoblastic niche and around blood vessels of the sinusoid niche. Moreover, our results are in accordance with recent data, which showed that subendosteal region is also rich in blood vessels suggesting that endothelial cells might be part of the subendosteal niche forming together a common niche where both, endosteum and sinusoids contribute to hematopoiesis.

The osteopontin expression in MSC close to endosteum is an indicative feature of their osteogenic differentiation capacity as a member of the osteoblastic niche. It has been previously reported that osteopontin expression is essential for HSC maintenance. In our model, its expression by human MSC near endostium could represent their intend to form an osteoblastic niche, trying to restore osteoblastic damage secondary to irradiation. Regarding SDF1 expression, previous data indicate that in BM SDF1 could be shown by both osteoblasts and endothelial cells. Our data are in this line showing a population of human MSC in the bone esdosteal surface that express SDF1 and another population distributed around blood vessels where endothelial cells are located. The expression of SDF1 by cells close to blood vessels could emphasize their affect as a chemotactic factor for HSC.

Nevertheless we could not find human hematopoietic cells close to these human stromas, they were proliferating in colonies or groups of human hematopoietic cells but their distribution did not show any relationship with human MSC. This data, along with the low number of human MSC detected, could indicate that these cells contributed to restore mice BM niche after irradiation but not to create independent human niches and probably their mechanism of action was based on paracrine mechanisms.

In summary, results of the present study show that chemotherapy damaged stromas can be restored *in vitro* by TP-MSC, which were able to lodge in cases where there is considerable stromal damage and they could restore SDF1 and osteopontin expression by a paracrine mechanism. In addition in our animal model human MSC were detected in murine BM when they were delivered by IB route, but only in the injected femurs and they can also favor the hematopoietic engraftment by their expression of SDF1 and osteopontin.

## Materials and Methods

### MSC isolation, expansion and characterization

Human MSC were isolated from BM cells from 20 healthy donors (HD) (8 males/12 females). Their median age was 41 years (range 30 to 61 years). In all cases written informed consent was previously obtained according to institutional guidelines in accordance with and approved by the local Ethics Committee of the Hospital Universitario de Salamanca. Ten to twenty milliliters of BM aspirate were taken from the iliac crest under local anesthesia according to standard institutional procedures. Mononuclear cells (MNC) from BM were obtained by a density gradient centrifugation (Ficoll-Paque, GE Healthcare Bio-Sciences, AB, Uppsala, Sweden) and cultured in standard culture medium as previously described [38].

For osteogenic differentiation, MSC were plated at 5×10^3^ cells/cm^2^ in a 9.6 cm^2^ slideflask (Nunc, Roskilde, Denmark), expanded up to 80% confluence and then further incubated in specific osteogenic medium (NH Osteodiff Medium; Miltenyi Biotec, Bergisch Gladbach, Germany). The medium was replaced every 3 to 4 days. After 10 days, cultures were washed, fixed and alkaline phosphatase activity was assessed by NBT/BCIP solution staining (Nitroblue tetrazolium chloride/5-bromo-4-chloro-3-indolyl-phosphate) (Roche, Basel, Switzerland), following manufacturer's recommendations.

For the adipogenic differentiation assay, 80% confluent MSC, seeded previously in a slideflask, were subsequently incubated in adipogenic medium (NH Adipodiff Medium; Miltenyi Biotec). The medium was changed twice a week for 21 days. Then, cells were washed, fixed and adipogenesis was measured by the accumulation of neutral lipids in fat vacuoles, stained with Oil-Red-O solution (Certistain Merck KGaA, Darmstadt, Germany).

For chondrocyte differentiation, 5×10^5^ cells were placed in a 15-mL polypropylene tube (Corning Incorporated, Corning, NY, USA) and centrifuged. The pellet was cultured at 37°C and 5%CO2 in 500 mL chondrogenic medium (NH Chondrodiff Medium; Miltenyi Biotec). Medium was changed every 3 to 4 days. After 21 days of culture, pellets were embedded in paraffin, cut into 5 µm sections and chondrocyte differentiation was evaluated by toluidin blue staining (Chemicon International, Hofheim, Germany).

For immunophenotypic analyses, cells were harvested and resuspended in PBS. MSC were incubated for 15 minutes with fluorescein isothiocyanate (FITC)-conjugated CD90, FITC-conjugated CD44, FITC-conjugated CD34, phycoerythrin (PE)-conjugated CD73, PE-conjugated CD14, PE-conjugated CD166, phycoerythrin-cyanine 5 (PC5)-conjugated Anti-HLA-DR, PC5-conjugated CD45, and PC5-conjugated CD19 (all from Becton Dickinson Biosciences, San Jose, CA, USA) and allophycocyanine (APC)-conjugated CD105 (R&D Systems, Minneapolis, MN, USA). Sample acquisition was performed in a FACSCalibur flow cytometer (Becton Dickinson Biosciences). Calibration of the instrument was performed prior to data acquisition using previously well-established protocols. CellQuest software program (Becton Dickinson Biosciences) was used for the acquisition of 50,000 total events. Data were analyzed using the Infinicyt software (Cytognos, Salamanca, Spain), as previously described [39].

### Chemotherapy

Using a previously established method, etoposide was added to induce chemotherapy-stromal damage [40]. Etoposide (Sigma-Aldrich, Steinheim, Germany) was reconstituted in dimethyl sulfoxide (DMSO) (Sigma-Aldrich, Steinheim, Germany) at a concentration of 20 mg/ml and stored at −20°C. Etoposide was diluted in appropriated medium prior to use. The chemotherapeutic drug doses were chosen to approximate doses described in clinical settings of transplantation and high dose chemotherapy doses [40], [41].

### *In vitro* MSC lodging assay

Third-passage confluent MSC were used as a feeder layer in 12.5-cm^2^ culture flasks (Falcon, Becton Dickinson, Le Pont De Claix, France) and cultured with LTBMC medium for 3 weeks to induce stromal differentiation, as previously described [42].

In order to test whether TP-MSC could lodge onto damaged stromas or if they have a paracrine effect, MSC from a different sex donor were obtained and used as TP-MSC for a second inoculum in a separate set of experiments. These experiments were performed in both control and etoposide-treated cultures at different concentrations (50 and 100 µM). In parallel, the same cultures were performed by adding only TP-MSC supernatant for paracrine effect studies. Cultures receiving a second inoculum of MSC were analyzed after five days and two weeks to study short- and long-term lodgment, respectively.

For TP-MSC lodging analyses, cells were harvested and fixed for FISH studies using sexual chromosomes probes following standard procedures as described elsewhere [43]. Briefly, slides containing fixed MSC were denatured in pepsin solution for 10 min at 37°C and dehydrated in sequential incubations of 70%/85%/100% ethanol at room temperature. A 10 µl hybridization mixture consisting of X (orange) and Y (green) probes (Vysis-Abbot Laboratories, Downers Grove, IL, USA) was prepared for each 24 mm^2^ coverslide. Samples were incubated for 6 min at 75°C and 20 hours at 37°C. After hybridization, slides were washed in 50% formamide in 2× SSC (0.3 M NaCl and 0.03 M Na citrate) pH 7.0 at 46°C for 5 min and once in 2× SSC. After washes, 1 µg/ml 4′, 6-Diamidine-2′ -phenyl indole, dihydrochloride (DAPI) in PBS was added and the cells were incubated at room temperature for 5 min. Slides were viewed with a Leica DMI6000B fluorescence microscope (Leica Microsystems GmbH, Germany) equipped with camera system. At least 100 nuclei were scored for each probe and results were expressed as percentage of cells from second MSC inoculum.

To determine whether MSC were acting throughout a paracrine effect, cultures receiving TP-MSC supernatant were studied during the entire culture period by assessing MSC confluence. Indirect cell counts were made to determine confluence.

### *In vitro* immunofluorescence analysis

To establish whether molecules secreted from TP-MSC could improve SDF-1α and osteopontin expression, either fresh medium or TP-MSC supernatant was added to etoposide-treated or untreated stromas (n = 8). In order to test SDF-1α and osteopontin expression immunofluorescence was used following previously described methods [44]. Two sets of cultures were analyzed: stromas from MSC untreated or treated with etoposide. To establish whether molecules secreted from normal MSC could improve SDF-1α and osteopontin expression in damaged stromas, either fresh medium or TP-MSC supernatant for 48 h (short-term) or 3 weeks (long-term) were added to cultures. The expression of SDF-1α and osteopontin was measured, as well as the morphology of stromal cells, to test the effect of TP-MSC supernatant (paracrine effect of MSC) on damaged stromas. As primary antibodies, rabbit polyclonal anti-human stromal-derived factor (SDF-1α) (Abcam, Cambridge, UK) and mouse monoclonal anti-human osteopontin (Abcam) were used. Cells were fixed with 4% paraformaldehyde and stained for the previously described primary antibodies. As secondary antibodies, rabbit anti-mouse Cy3 for osteopontin detection and goat anti-rabbit Cy2 for SDF-1α analysis were used. Finally, slides were mounted and observed with a Leica DMI6000B fluorescence microscope equipped with a camera system, using the appropriate filter for each fluorescence. Images were captured and then analyzed using Leica Application Suite 2.02.

### Western blot

Western blot was performed to confirm the results of the previous analysis of osteopontin expression following methods described elsewhere [45]. Three weeks after etoposide treatment, cells were washed with PBS (Gibco, Invitrogen, Paisley, UK) and lysed in ice-cold lysis buffer (140 mM NaCl, 10 mM EDTA, 10% glycerol, 1% Nonidet P-40, 20 mM Tris pH 7.0, 1 µM pepstatin, 1 µg/mL aprotinin, 1 µg/mL leupeptin, 1 mM sodium orthovanadate). Fifty micrograms of the protein samples were subjected to 12% SDS/PAGE and blotted onto PVDF membrane (Millipore). Membranes were incubated with mouse anti-osteopontin (1∶500) and mouse anti-tubulin (1∶100) (Santa Cruz Biotechnology, Santa Cruz, CA, USA). Membrane-bound first-step antibodies were reacted with horseradish peroxidase-conjugated anti-mouse (GE Healthcare, Amersham, UK) and bands were visualized with a luminol-based detection system with p-iodophenol enhancement [45].

### RT-PCR analysis

Real-time quantitative polymerase chain reaction (RQ-PCR) was performed to confirm the previous analysis of osteopontin and SDF1 expression in four independent sets of experiments [46]. RNA was isolated with the RNeasy Mini Kit (Qiagen, Valencia, CA, USA) and quality and quantity were assessed with NanoDrop. The retrotranscription reaction was performed with a High Capacity cDNA Reverse Transcription Kit (Applied Biosystems Foster City, CA, USA) according to the manufacturer's recommendations. RQ-PCR was carried out on cDNA using Assays-on-Demand gene expression mixes specific for osteopontin and SDF1 (Hs_00167093 and Hs_00171022, respectively) and the TaqMan Fast Universal PCR master mix (Applied Biosystems). Reactions were carried out in a StepOnePlus Real-Time PCR System using 20 ng of cDNA in a final volume of 10 µL. RQ-PCR amplification of the ABL gene was used to assess RNA quality and quantity and to normalize gene expression in the experiments. The relative quantification of gene expression was performed using the cycle threshold increment method.

### Animals

Six weeks NOD.CB17-*Prkdc^scid^*/NcrCrl mice were pursached from Charles River Laboratories (Barcelona, Spain), housed in microisolator cages and maintained under sterile conditions in the animal facility of the University of Salamanca. All procedures were used, following the Spanish and European Union guidelines (RD 1201/05 and 86/609/CEE, respectively) and after the approval of the local Bioethics Committee of the University of Salamanca (Reg.N° 201100007927).

### *In vivo* MSC engraftment

The NOD/SCID mouse xenotransplant model was established as previously reported, with slight modifications [47]. A total of 64 mice were used for this experiment. Eight weeks old mice were exposed to 300-cGy total body irradiation from a CS source (Gammacell-200, Nordion International, Ottawa, ON, Canada). Six to eight hours after irradiation, the animals were anesthetized with a mixture of ketamine (90 mg/kg; Imalgene 500, Merial, Lyon, France) and xylazine (10 mg/kg; Rompun 2%, KVP Pharma, Bayer Healthcare, Kiel, Germany) for transplantation. In order to test the role of the injection site of hematopoietic cells and MSC in hematopoietic engraftment, human HSC cells were intravenous (IV) or intrabone marrow (IB) administrated while human MSC were exclusively IB injected under the following conditions: 1) 2×10^6^ IV CD34+ cells; 2) 2×10^6^ IV CD34+ cells and 5×10^5^ IB MSC; 3) 2×10^6^ IB CD34+ cells; 4) 2×10^6^ IB CD34+ cells and 5×10^5^ IB MSC and sixteen mice were included in each experimental group. In all cases, CD34+ cells and MSC were obtained from a sex-mismatched donor. Intravenously injected cells were resuspended in a 200 µl of PBS and slowly injected by the tail vein. For IB injection, a 27-gauge needle was inserted into the joint surface of the right femur, and human cells were injected into the BM cavity in a total volume of 20 µl. Six weeks after transplantation animals were killed. Half of mice femurs from each group were used for MSC expansion and subsequent human MSC detection by FISH. The other half was fixed for immunohistochemistry analysis. Both will be explained next. Hematopoietic engraftment has been previously described in part one.

### Human MSC detection in murine BM by FISH

MNC obtained after 6 weeks from both femurs were seeded in 12-wells culture plates (Corning) under the same conditions previously described for human MSC expansion. For human MSC detection, cells were harvested after the second passage and stained for FISH using human sex chromosome probes as previously described for *in vitro* assays. Briefly, a 10-µl hybridization mixture consisting in X (orange) and Y (green) probes (Vysis-Abbot Laboratories, Downers Grove, IL, USA) was prepared for each 24 mm^2^ coverslide. Slides were viewed with a Leica DMI6000B fluorescence microscope. Total nuclei were scored; results were expressed as total number of human MSC detected by sample.

### Immunohistochemistry analysis in murine femurs

We used immunohistochemistry to test whether SDF-1α and osteopontin were modified after healthy MSC addition. Murinefemurs were fixed, paraffin embedded and 5 µm thick tissue sections were placed on glass slides. Paraffin sections were deparaffinized and endogenous peroxidase activity was blocked by immersing the sections in 3% hydrogen peroxide for 5 min. The sections were then incubated with 5% normal donkey serum and bovine serum albumin to block nonspecific binding. As primary antibodies, the previously described anti-SDF-1 and anti-osteopontin antibodies were used. In order to detect if expressing cells were from human origin, also a primary antibody for human mitochondria detection has been used in sequential sections. As secondary antibodies, biotin-conjugated donkey anti-mouse IgG for human osteopontin and mitochondria detection and donkey anti-rabbit IgG for SDF-1α analysis were used (both from Jackson ImmunoResearch, West Grove, PA, USA). The sections were then incubated with avidin-biotin-peroxidase complex ABC Elite Kit PK-6100 (Vector Laboratories, Burlingame, CA, USA) for 30 min at room temperature. After washing with PBS brown pigmentation was produced by treatment with 3,3′-diaminobenzidine (DAB) (Sigma, St Louis, MO, USA)

Finally, slides were mounted and observed with an Olympus BX41TF microscope (Olympus Optical Co., Tokyo, Japan).

### Statistical analysis

Medians and ranges were calculated for each variable. The non-parametric Mann-Whitney U-test was used to estimate the significance of the differences between groups. Differences were considered to be significant for values of p<0.05. All statistical analyses were done with SPSS 17.0 (Chicago, IL, USA).
